# Plasmacytoid Dendritic Cells: From Heart to Vessels

**DOI:** 10.1155/2010/430318

**Published:** 2010-09-22

**Authors:** Rosalinda Sorrentino, Silvana Morello, Aldo Pinto

**Affiliations:** Pharmaceutical Science Department, University of Salerno, 84084 Fisciano, Italy

## Abstract

Cardiovascular diseases, formerly only attributed to the alterations of the stromal component, are now recognized as immune-based pathologies. Plasmacytoid Dendritic Cells (pDCs) are important immune orchestrators in heart and vessels. They highly produce IFN type I that promote the polarization of T cells towards a Th1 phenotype; however, pDCs can also participate to suppressive networks via the recruitment of T regulatory cells that downmodulate proinflammatory responses. pDCs populate the vessel wall layers during pathological conditions, such as atherosclerosis. It is thus clear that a better identification of pDCs activity in cardiovascular diseases can not only elucidate pathological mechanisms but also lead to new therapeutic approaches.

## 1. Introduction

Cardiovascular diseases, formerly only attributed to the alterations of the stromal component, are now recognized as immune-based pathologies. [1]. Only in the late 1980s, studies highlighted the link between the inflammatory response and the immune system in cardiovascular diseases [1, 2]. Indeed, inflammation is a component of many vascular disorders such as aortic aneurysm, giant cell arteritis, Takayasu's Disease, Kawasaki Disease, and atherosclerosis [1, 2]. 

The cardiovascular system can be compromised by hemodynamic factors, such as the high blood pressure, by the thickness of the aortic sinus and aorta [1, 2]. However, the immune system contributes to the exacerbation of cardiovascular pathologies, such as atherosclerosis, heart failure, arteritis, and aortic aneurysm [1]. Any kind of insult to the cardiovascular system can induce the release of proinflammatory cytokines and chemokines and facilitate the recruitment of immune cells, such as dendritic cells, macrophages, and T cells, which promote the inflammation with the release of a variety of inflammatory mediators [1, 2]. In turn, the stromal cells, such as smooth muscle cells and endothelial cells, also participate in this process either by expanding the extracellular matrix or by releasing other proinflammatory molecules. This tends to exacerbate inflammation that alters vessels and heart functionality [1, 2]. 

## 2. Conventional Dendritic Cells (cDCs) and Plasmacytoid Dendritic Cells (pDCs)

Dendritic cells (DCs) are professional Antigen Presenting Cells (APCs), which process and present antigens to naïve lymphocytes, B cells, Natural Killer (NK), and NKT cells [3]. DCs are identified in two specific subsets, conventional (cDCs) and plasmacytoid DCs (pDCs) [4, 5]. In contrast to humans, mice are characterized by many different DCs subsets as determined by the cluster differentiation markers [6]. Mouse DCs are identified as CD11c^high^ CD11b^low^ but can also be discriminated by the expression of CD4+ CD8*α*+ and CD4-CD8*α*- [6, 7]. 

Human pDCs are CD4+ CD45RA+ IL-3*α*R (CD123)+ ILT3+ ILT1- CD11c^low/−^ cells [6]. Two additional surface markers on human pDCs are represented by BDCA-2 and BDCA-4, that correspond to the murine mPDCA-1, restricted to the peripheral blood and bone marrow-derived pDCs [6]. Mouse pDCs share most of the morphological and phenotypical features with the human counterpart; however, murine pDCs are distinguished as CD11c^low/−^ Gr-1^+/intermediate^ B220+ 120G8+ cells [6, 7]. The recognition of the cluster differentiation proteins is actually very important not only to distinguish pDCs from cDCs and other cell types but also for the isolation of these cells.

Mouse pDCs have been identified in lymphoid organs but also the liver, the lung, the heart, the vessels and the skin although their proliferation rate is very low during physiological conditions [7, 8]. Instead, human pDCs populate primary, secondary and tertiary lymphoid organs (aggregates/follicles), the liver and the blood [9]. During pathological conditions, pDCs migrate from the bone marrow or the circulation to the damaged tissue through the high endothelial venules [7]. CD62L expressed on pDCs ligate L-selectin ligands expressed on endothelial and other stromal cells [6]. The repertoire of chemokine receptors on pDCs is higher expressed than those on cDCs [6]. CXCR3, highly expressed under interferon (IFN) *γ* production, binds to CXCL19 and CXCL10 and is highly required by pDCs to migrate into inflamed lymph nodes [10, 11]. Additional chemokine receptors expressed by pDCs are CCR1, CCR2, and CCR5 that bind to CCL2, CCL3, CCL4, and CCL5; they express CXCR4, implicated in pDCs migration, and CCR7 for the constitutive chemokines CXCL12 and CCL21 [10, 11]. 

The recognition of some pathogen-associated molecular patterns (PAMPs) and/or danger-associated molecular patterns (DAMPs) by pDCs promotes their mobility into the circulation up to the damaged tissue [12, 13] whereas cDCs can also recognize the antigens into the tissue and then present them into the lymphoid organs. One of the functional consequences is that pDCs are not recognized as APCs [13], as in the case of cDCs, but they can polarize T cells towards a Th1 or immunosuppressive phenotype [13, 14]. 

## 3. Activation of Plasmacytoid DCs (pDCs)

pDCs are defined as professional interferon- (IFN-) producing cells (IPCs), a characteristic that depends on the expression of some cytoplasmic receptors, such as TLRs, that promptly detect DNA or RNA from viruses and bacteria [14]. pDCs and cDCs have a different repertoire of TLRs expression. Human and mouse cDCs can express TLR1, 2, 3, 4, 5, 7, and 8 [15], while pDCs uniquely express TLR7 and TLR9 in the endosomes [16], which sense nucleic acids [14]. Many studies have shown that the exposure of pDCs to TLR7 or TLR9 agonists (imiquimod and CpG-ODN, respectively) can lead to the production of IFN**α** and proinflammatory cytokines, such as TNF**α** and IL-8, essential to strengthen pDC-mediated T cell stimulation [17]. Very importantly, IFN**α** modulates several aspects of the immune system, including pDCs survival, cDCs differentiation, modulation of Th1 and CD8+ T cell responses, crosspresentation, upregulation of MHC and costimulatory molecules, activation of NK cells, and induction of primary antibody responses [17]. 

pDCs activation can also lead to the production of IL-12p70 [14, 17], IL-1*β*, and IL-6 [14]. Furthermore, it is of very recent discovery that pDCs may mediate the release of IL-10 [18]. However, an outstanding group proved that these cells do not directly produce IL-10 [19].

## 4. pDCs are Bridging Cells between the Innate and the Adaptive Immunity

The production of IFN type I renders pDCs the bridge between the innate and the adaptive immune system. IFNs type I (IFN*α* and IFN*β*) are released upon viral infections [17] and also during some cardiovascular diseases, such as atherosclerosis [20]. Upon activation, pDCs, rather than cDCs, produce higher amounts of IFN*α* that, in turn, amplifies the production of the same cytokine or IL-12p70 from cDCs and NK cells [17, 21], ending with the activation of the adaptive immune system. Thus, activated pDCs can directly or indirectly induce T cell IFN*γ* production, promote Th1 polarization [13, 14], and induce cytotoxic T lymphocyte- (CTL-) mediated responses [13] as well as proliferation and survival of T cells. 

However, very recent studies demonstrate that pDCs can also lead to the synthesis of a tolerogenic enzyme, indoleamine-2, 3-dioxygenase (IDO) [22]. IDO is highly present in human aortic smooth muscle cells and atheroma [23], where it is associated to the tolerogenic inducible costimulator ligand (ICOS-L), implicated in the regulatory T cells network [23, 24]. To note, the increased synthesis of IDO is associated to the autocrine effect of IFN type I on pDCs upon, for example, TLR9 activation and CD200R ligands stimulation [14, 24]. Very interestingly, pDCs can recruit T regulatory (Treg) cells through direct and indirect interaction via the PD-L1/PD-1 axis [25, 26] and immunosuppressive cytokines, such as IL-10 [26]. 

It is obvious, then, that pDCs can have a dual role, immunostimulant or immunosuppressive. A possible explanation of this difference may simply be correlated to the physiological negative feedback that occurs when an excessive cytotoxic response can damage the host, or to the pathological microenvironment. For example, the prostaglandin E_2_ (PGE_2_), proinflammatory mediator highly relevant in cardiovascular pathologies, can inhibit IFN*α* production and the Th1 polarization induced by pDCs [27]. However, more studies are needed in this regard to prove that PGE_2_ alters pDCs activity during a cardiovascular disease. 

Upon these findings, it seems very important that the accumulation of pDCs into tissue may influence T cell polarization and more importantly participate in the resolution of the injury. 

## 5. Plasmacytoid Dendritic Cells (pDCs) and their Role in Cardiovascular Diseases

Two types of DCs, cDCs and pDCs, have been identified in the atherosclerotic lesions, giant arteritis, and Kawasaki Disease [1, 2, 28, 29]. DCs are present in the normal and pathological intima [30, 31] and adventitia [32, 33]. This strategical position allows DCs to be in a surveillance mode to recognize antigens and then move to the draining lymph nodes to ‘present’ to T cells. 

Immature DCs (CD80/CD86 low-MHC II low) are physiologically located in the adventitia and media of blood vessels [28]; however, any type of insult, ranging from pathogen-derived or host-derived, can facilitate the migration of DCs from the adventitia/media to the intima [28]. The interaction between DCs and endothelial cells can then promote the recruitment of other immune cells [1, 2, 28]. The activation of the adaptive immune system may influence the resolution of the injury or the prolonged immune attack, which facilitates chronic inflammatory conditions, deleterious for the cardiovascular system. 

Very recent data from Weyand's group indicate that both cDCs and pDCs populate the human atherosclerotic plaques [34, 35]. cDCs physiologically reside in the adventitia and media of the vessel wall [1, 2, 28]. The recognition of antigens renders these wall-embedded cells essential for a first line of defence and surveillance. The migration of cDCs to the lymphoid organs facilitates pDCs to migrate towards the damaged tissues. In turn, pDCs can migrate to the draining lymph nodes where they can polarize T cells [12, 14]. 

pDCs are localized in the arterial and aortic sinus of atherosclerotic plaques in humans and mice [20, 33], concentrating into the unstable shoulder region of the plaques. The production of IFN type I leads to a 10-fold increase of TNF-related apoptosis-inducing ligand (TRAIL) that facilitates the apoptosis of vascular smooth muscle cells (VSMCs) laden with oxysterols (plaque resident cells) [20]. On the other hand, pDCs can modulate the generation/recruitment of CD4+CD25+Foxp3+ Treg cell that play an important role in the formation of atherosclerotic lesions in mice [36]. Treg hamper the Th1 immunity, avoiding deleterious invasive damages, but at the same time they impede the immune system to combat the cardiovascular risk. Th1 responses can be initiated by IFN type I whereas T regulatory pathways are initiated by IL-10 and/or TGF*β* [37]. pDCs release IFN type I but can also promote the synthesis of IL-10 and TGF*β* via their regulatory patterns such as IDO and ICOSL [23–25]. 

Similarly, excessive numbers of pDCs are observed in the blood of heart failure patients [38]. A very interesting paper has recently been reported showing that the deficiency of Interleukin-1 receptor-associated kinase-4 (IRAK- 4), downstream of MyD88-dependent TLRs signalling, avoids the myocardial infarction [39]. This effect is associated with the reduction of CD11c positive infiltrating cells. cDCs and pDCs are identified by this membrane integrin (CD11c). Although the role of cDCs in this mouse model of infarct is fundamental, it is also to note that pDCs may be as well. IRAK4 is implicated in all MyD88-dependent TLRs signalling, among which TLR7 and TLR9. It is not to exclude thus that the activation of these receptors in pDCs could be responsible for the myocardial infarction together with cDCs. But, the potential role of pDCs in this pathology is far from clear. 

Another important finding is the localization of pDCs in human and mouse Kawasaki Disease and giant arteritis samples [28]. So far, it is still not known what is the mechanism underlying the presence of pDCs in these pathologies; however, pDCs correlate with the presence of cDCs and T cells, leading to a possible functional role in the coronaries, such as T cell polarization [28]. 

## 6. Conclusions and Remarks

pDCs are IFN-producing cells which could mediate a Th1-like and T cytotoxic response; but also participate in the immunosuppressive network. 

Actually, there is no evidence that explains the vascular presence of pDCs. However, we could speculate that pDCs can be activated by circulating PAMPs and/or DAMPs, which would promote their localization to the inflamed locus such as in the case of the atherosclerotic plaques [20, 33]. pDCs activation can then potentiate cDCs activity [20] or alter adaptive immunity by inducing either Th1-like immunity or an immunesuppressive adaptive immunity. The microenvironment is essential to determine the capability of pDCs to produce IFN type I. Thus, the identification of their role, and most of all, the connection between their phenotype and the microenvironment may lead to new potential therapeutic approaches for cardiovascular-related pathologies. 
